# ‘It’s not really that funny’: Young people’s experiences of colourist humour

**DOI:** 10.1177/00380385261446254

**Published:** 2026-07-03

**Authors:** Aisha Phoenix, Nadia Craddock, Annabel Wilson, Jamila Thompson

**Affiliations:** 1 King’s College London, UK; 2 UWE Bristol, UK; 3 University of Oxford, UK

**Keywords:** adolescence, colourism, ethnic teasing, humour, intersectionality, jokes, school, white supremacy

## Abstract

Colourism is prejudice that penalises people the darker their skin is and the further their features are from those associated with whiteness. This article explores how adolescents in the UK use humour in ways that perpetuate colourism, particularly targeting those with dark skin. Drawing on reflexive thematic analysis of 130 interviews with minoritised ethnic students aged 13–18 years in London and Bristol, we argue that while colourist humour is often presented as harmless, it reproduces racist and colourist tropes that can dehumanise and harm those targeted and bystanders. Colourist humour was used to mark and test the boundaries of friendships and served to maintain racialised hierarchies, but it was also perpetuated by students with dark skin. While this may indicate that colourism is being internalised, our research suggests that this is not the whole story. Colourist humour is one way in which young people negotiate the harmful reality of everyday colourism.

## Introduction

Colourism is prejudice in which people face penalties the darker their skin, and the more their features differ from those associated with whiteness (Phoenix and Craddock, 2024). It shapes multiple areas of life, including education where skin shade is linked to unequal outcomes. USA research has found that lighter skin is associated with higher levels of attainment among Asian American adolescents (Ryabov, 2016), while darker skin is linked with fewer years of schooling for Black and Latino students (Hunter, 2016). Research also indicates that Black students with dark skin are subjected to harsher discipline than peers with light skin (Keyes and Crutchfield, 2020).

Understanding how colourism operates in schools is important in addressing colourist inequalities (Keyes and Crutchfield, 2020). We argue that a key but under-examined mechanism through which minoritised ethnic adolescents are subjected to colourism is humour in the form of colourist banter, teasing and jokes. Humour offers a means of signalling affiliation and negotiating social boundaries (Goffman, 1959). It can have positive effects, but can also be used to tease, bully and harass (Butler, 2023). While much research has examined racist humour and ethnic teasing (e.g. Benner et al., 2024; Caparoso and Collins, 2015; Douglass et al., 2016), far less has explored humour rooted in colourism. Consequently, little is known about how colourist humour shapes young people’s everyday experiences, relationships and wellbeing.

In this article, ‘colourist humour’ refers to what participants described as jokes, teasing and ‘banter’ directed at minoritised ethnic adolescents because of their dark skin shade and/or phenotypical features not associated with whiteness. We adopt this term rather than racialised or racist humour to foreground humour based on phenotypical features rather than solely on racialised group. While colourism and racism are closely interconnected, colourism is distinctive in that it can be perpetuated both within and across racialised groups (Dixon and Telles, 2017). Further, colourism can intensify experiences of racism. Minoritised ethnic people are more likely to be subjected to racialised discrimination the further their features are from those associated with whiteness (Brown et al., 2023; Phoenix and Craddock, 2024).

### The study

The data presented here come from a larger mixed methods study of understandings and experiences of colourism among adolescents in the UK. This article addresses three main questions:

1. How does colourist humour affect minoritised ethnic adolescents in UK schools?
2. How do adolescents understand and negotiate colourist humour?
3. How do racialisation and skin shade affect adolescents’ experiences of colourist humour at school and in everyday life?

Our findings show that colourist humour was a pervasive feature of school life and that adolescents with dark skin were most frequently identified as targets, sometimes teased by peers of a similar skin shade. Participants described colourist humour as more acceptable than racist humour, particularly when it occurred within the same racialised group. Although colourist jokes were frequently framed as light-hearted banter, they demeaned minoritised ethnic adolescents, including those who participated in the joking themselves.

Colourist humour exposes a limitation in theorisations of ethnic teasing that are not attentive to the role of phenotype. We argue that colourist humour helps normalise and sustain colourist and racist hierarchies by reproducing white supremacy (cf. Pérez, 2022), a system in which power and resources are controlled by White people; White people are positioned as superior; and racialised inequality is perpetuated through institutions and everyday interactions (Ansley, 1988–1989).

Pérez’s (2022) concept of ‘amused racial contempt’ maintains that humour can be used to put down people deemed to belong to an inferior racialised group and to position those making the jokes as belonging to, or in alliance with, a superior group. We extend Pérez’s conceptualisation to argue that students employed ‘amused colourist contempt’ to belittle peers with dark skin or features that did not resemble those associated with whiteness, to position themselves higher up colourist hierarchies by comparison and/or boost their social standing by aligning themselves with those of a higher status. The fact that colourist humour was perpetuated by minoritised ethnic people of different skin shades, as well as White people, helped to normalise the discrimination, making it harder to challenge and resist. Furthermore, focusing on phenotype, rather than ethnicity, facilitated the construction of colourist discrimination as just banter. We argue that colourism is best understood through the prism of intersectionality, which facilitates an examination of how a person’s social location, such as their gender, skin shade and ethnicity, affects their experiences (Crenshaw et al., 2024).

## Literature review

### Colourism research in the UK

UK colourism research remains limited and primarily focused on adults. Qualitative studies have examined colourism in relation to Black men’s identities and experiences (Phoenix and Craddock, 2022), family relationships and intergenerational dynamics (Phoenix and Craddock, 2024), intimate relationships and desirability (Phoenix and Craddock, 2024) and wellbeing (Spratt, 2024). These studies highlight how colourism is linked to everyday interactions and life chances. Quantitative research has begun to document associations between experiencing colourism and wellbeing in adults (Craddock et al., 2023a, 2023b) and more recently in adolescents (Craddock et al., 2025a, 2025b). However, there is a lack of in-depth analysis on how colourism operates for adolescents in everyday settings such as schools, or on the role of humour in sustaining colourist hierarchies.

## Colourism and disparagement/racial humour

A small number of studies have acknowledged colourist humour within broader analyses of colourism and racialised interaction. In qualitative UK research, Phoenix and Craddock (2022) briefly highlight colourist jokes among Black men and their social and psychological effects. In the USA, Guzman (2020) examines colourist humour within elite Black men’s joking practices, while English and colleagues (2020) note how such jokes shape Black adolescents’ perceptions of skin shade and identity. However, to our knowledge, no research has centred on colourist humour or teasing.

Given the limited literature on colourist teasing, it is useful to draw on broader theories of ethnic or racialised teasing (hereafter ethnic teasing) to contextualise the current research. Teasing can be defined as ‘the intentional provocation of a target individual around a topic important to them with some level of playfulness’ (English et al., 2020: 2). Ethnic teasing refers to social interactions in which humour is used to reference a person’s ethnicity or racialisation, often conveying prejudice under the guise of joking. Such interactions may appear innocuous, especially among peers, but can perpetuate stereotypes and microaggressions (Douglass et al., 2016). The forms ethnic teasing takes include racist name calling, phrases, rhymes, songs, or jokes (Caparoso and Collins, 2015) and it is considered a form of ‘disparagement humour’ (Ford and Ferguson, 2004).

Disparagement humour trivialises discrimination by normalising discriminatory attitudes, particularly among those who hold prejudicial views (Ford and Ferguson, 2004). It also pressures targeted individuals to adopt a ‘non-serious mindset’, forcing them to accept or dismiss the joke in a light-hearted way (Ford and Ferguson, 2004). Doing so may reflect an internalised acceptance of discriminatory norms, a belief that in certain instances humour is being used to subvert stereotypes (Ford and Ferguson, 2004), or a lack of power to resist. Either way, while humour may downplay the seriousness of racist remarks, ethnic teasing reinforces racialised hierarchies and places the burden on the target not to react to the discrimination (Benner et al., 2024) or risk being ‘discredited’ and/or accused of ‘taking things too seriously’ (Trindade, 2021: 72).

## Sociology of humour: Racist jokes and white supremacy

Sociological analyses of humour have shown that joking is a powerful mechanism through which racialised hierarchies are normalised and reproduced in everyday life. In his exploration of how racist humour perpetuates white supremacy, Pérez (2022: 16) argues that ‘racial ridicule’ was commonplace in 19th- and early 20th-century USA, where ridiculing Black people enabled White people to feel ‘racially superior’. His concept of ‘amused racial contempt’ encapsulates both this and contemporary forms of humour being used to perpetuate white supremacy. Drawing on this framework, we conceptualise colourist humour as a related but distinct form of amused contempt, one that operates through gradations of skin shade and phenotypical difference, and is enacted not only across racialised groups but also within them, including among minoritised ethnic adolescents in school contexts.

## Impact of colourism and disparagement/racial humour on adolescents

Adolescence is an important developmental period in which young people are especially sensitive to peer acceptance and group norms (Herd and Kim-Spoon, 2021). Ethnic teasing during adolescence often occurs among close peers and friends (Douglass et al., 2016), creating social pressure to accept or tolerate such ‘jokes’ and increasing the likelihood that they are dismissed as harmless (Benner et al., 2024; Douglass et al., 2016). However, increasingly, research demonstrates that ethnic teasing has negative consequences for adolescent wellbeing. Hearing racist jokes from friends has been linked to heightened daily stress, anger, anxiety and depression among ethnically diverse adolescents in the USA (Benner et al., 2024). Similarly, experiencing or witnessing ethnic teasing has been associated with both increased social anxiety among ethnically diverse adolescents in the USA with high baseline anxiety (Douglass et al., 2016) and more depressive symptoms among Black American adolescents (English et al., 2020). Experiences of colourism, including colourist teasing, is associated with worse wellbeing among Black and South Asian adolescents living in the UK (Craddock et al., 2025).

## Method

The article is based on the narratives of 130 minoritised ethnic students at secondary schools or sixth-form colleges in Bristol and London. These cities were selected based on their long-established, multi-ethnic populations. In the 2021 Census, 18.9% of Bristol residents and 46.2% of London residents identified as Asian, Black, Mixed or ‘other’ ethnic groups. Each participating school or college had a substantial proportion of minoritised ethnic students – up to 70% of the school or college population in Bristol and up to 90% in London (i.e. notably higher than the 35.4% average for secondary schools in England; School Census 2022/23).

Sixty-six of the students were in years eight or nine and 64 were in years 12 or 13. Fifty-seven of the students were Black (Black African, 41; Black Caribbean, 12; and both, four), 26 were Mixed Black-White, 28 were South Asian (Indian, 15; Sri Lankan, four; Bangladeshi, five; Bengali, two; and Pakistani, two) and 19 were of other minoritised ethnicities.

Ethical approval was obtained from King’s College London. Secondary schools and sixth-form colleges in Bristol and London were invited to participate via correspondence with school leadership. Once institutional agreement was obtained, advertisements were placed in school/college buildings and on electronic bulletins.

Parental consent was obtained for students under the age of 16. Students provided informed consent to participate in an interview after reading our information sheet, which detailed the project aims and terms of participation. Students also completed a demographic information form that included a question asking them to select their skin shade from five written options: very dark, dark, medium, light or very light. In-person, semi-structured interviews were then held at participants’ schools/colleges in 2023. These lasted 47 minutes on average.

Interviews, conducted by the first three authors, explored how skin shade affected experiences at school, at home and in community, religious or sport settings. At the beginning of each interview, we asked participants to discuss their appearances while making a memoji of themselves, which included selecting their skin shade on an iPad. This helped prepare participants to begin reflecting on how their racialised phenotypical features affected their experiences at school and beyond and it offered insights into how participants saw themselves. Interview questions included: ‘Do people at school get teased because of their skin shade?’ and ‘Do you think your skin shade affects how people treat you at school?’. After a dozen interviews, we observed that younger participants were reticent to talk about colourist teasing when asked whether they have ever noticed it, while older participants mentioned its frequency when they were younger. Therefore, we included additional prompts when interviewing younger students.

The narratives explored below were selected because they either illustrate points that were being made by numerous participants or they countered or complicated dominant perspectives. The 130 narratives analysed for this article reflect our participants’ diversity and our desire to investigate the experiences of minoritised ethnic students of different racialised backgrounds and genders at different points in their school career across nine schools or colleges in Bristol and London. Names were pseudonymised and identifying information was removed.

The dataset was analysed through an intersectional lens using reflexive thematic analysis, a theoretically flexible method of qualitative inquiry (Braun and Clarke, 2019). Analysis was led by AP, who read the transcripts multiple times to familiarise herself with the data and took an inductive approach to coding the transcripts, prioritising participants’ accounts. She identified patterns in the codes and developed candidate themes, which she checked by re-reading the transcripts, before all authors agreed the final themes.

### Positionality and reflexivity

Since researchers co-create data with participants and then create meaning from it, it is important to situate the researchers in this work (Braun and Clarke, 2019). AP is African Caribbean with dark skin and Afro hair. She was subjected to colourism growing up. NC is of mixed ethnicity with one Indian and one White parent. She has been aware of colourism and beauty ideals privileging light skin since childhood. AW has light skin and is of mixed ancestry. She grew up in a Caribbean household that actively reinforced colourist beauty ideals. JT is an African Caribbean researcher who experienced colourism at school and in her family. AP, NC and JT grew up in London and AW grew up in Bristol. As researchers of colour with personal experiences of colourism, our biographies and memories have informed each stage of the research process. For example, witnessing colourist humour when we were growing up gave us insights into how to elicit participant narratives about colourist humour and to feel at ease exploring participants’ experiences. Our positionality as minoritised ethnic women critical of colourism may have helped participants to feel comfortable discussing their experiences with us. As a research team, we used our intersectional epistemology and ‘embodied knowledge’ reflexively to inform our approach to the research and analysis, alongside our academic knowledge and disciplinary training (Shapiro, 1999).

## Colourist humour targeting young people

Eighty-nine percent (*n* = 115) of participants described colourist jokes at their current school, previous schools or both. A few mentioned colourist jokes beyond school, and about 10% (*n* = 13) either said there were no colourist jokes or did not give any examples. Two of these 13 volunteered examples of racist jokes that were not directly related to skin shade or phenotypical features. For example, Danielle described the racist way in which peers treated an Indian student who was not fluent in English: ‘They used to basically take the mick out of her, they used to literally treat her like a dog [. . .] they would like pet her on the head’.

Students highlighted several common skin shade jokes about Black young people with dark skin. There were also jokes about South Asian people with dark skin; however, these were mentioned less frequently, perhaps reflecting the greater number of Black students in the sample and the demographics of participating schools. Students described jokes likening those with dark skin to primates and mentioned jokes about other phenotypical features, such as Black people having ‘nappy’ hair, South Asian people being hairy, East or Southeast Asian people having small eyes and adolescents of various minoritised ethnicities having large noses.

One of the most common jokes about dark skin was along the lines of ‘Every time the light goes out, “I can’t see you, where are you?”’ (Gabriel, a 14-year-old Filipino boy with light skin), variations of which were repeated across participant accounts. Related comments were made by several participants, including the following examples that were mentioned by Black girls with dark skin: ‘We can only see your teeth’, ‘You’re so Black, you could hide in the dark’ and ‘Can’t see you under the bed’.

Young people said that colourist humour included students with dark skin being labelled ‘blick’, a derogatory term for someone with very dark skin (Dictionary.com, 2018). Abina, an 18-year-old young Black African woman with dark skin, defined it as: ‘darkest of dark, like my bag black’. Fawzia, a 13-year-old East African girl with dark skin, gave an example of boys making comments about a girl: ‘She’s got really dark skin and then they’re always saying, “Shut up you blick girl”, stuff like that’.

In pathologising dark skin through amused colourist contempt, students simultaneously normalised and privileged whiteness and, to a lesser extent, light skin. As Raby (2004) notes, whiteness confers material and structural advantage, serving as the norm against which minoritised ethnic people are othered. Drawing on research with teenage girls, Raby (2004: 375) argues that whiteness as a category was visible only temporarily and occasionally, with ‘white “culture”’ or privilege often rendered invisible. Although participants in our study occasionally mentioned making jokes about peers with white or light skin, most skin-shade humour focused on dark skin.

Some participants said that South Asian students with very dark skin were called ‘Black’ and allowed to say the ‘N-word’, which emphasised their darkness and the similarity between them and Black people, who are implicitly considered to be darker. For example, in response to the question ‘[W]ere there any jokes specifically around skin shade?’, Dilara, a 17-year-old Bangladeshi young woman with medium skin, said:

[B]rown people who are quite dark, even people who are like Tamil, who are quite dark, or Indian, like they get a lot of comments about how dark they are. [. . .] They say stuff like, ‘You can classify yourself as Black’, or like, ‘You have the N-word pass’.

Here, ‘amused colourist contempt’ serves to elevate the perpetrator’s status, while lowering South Asians with dark skin to the position of Black people in colourist and racialised hierarchies. There is a long history of Indians with dark skin being characterised as Black. Kullrich (2022) argues that in the 19th century, during Britain’s colonialisation of India, the White British elite constructed Indians with dark skin as Black and described them as the ‘N-word’.

Several participants discussed students calling those with dark skin, particularly Black people, monkeys, or likening them to primates. Many shared their experiences of being called a monkey or being subjected to simian animalistic dehumanisation couched in humour. For example, Ayaan, a 14-year-old Black African girl with medium skin, said that when monkeys featured in her English class:

[T]he two darker-skinned Black students in my class will be like ‘That’s you, that’s you’.

Interviewer: To each other?

Yes. As a joke.

Black people were the racialised group most often identified as making, and being the target of, colourist jokes, reflecting their positioning in colourist and racialised hierarchies (Phoenix and Craddock, 2022). Boys were described as perpetrating, and being subjected to, colourist jokes more than girls, which is consistent with the idea of joking as a ‘masculine practice’ boys have to negotiate (Peltola and Phoenix, 2022: 99). Tracie, a 14-year-old Black African girl with dark skin, said that peers call a classmate who is ‘big’ and ‘tall’ with dark skin a ‘gorilla’:

[B]ecause he’s tall, he’s got more fat on his body, and he’s dark skinned, so they say he’s a gorilla [. . .]. Doing gorilla motions towards him, banging their chest. And all of that, which is very dehumanising to be honest.

Her narrative suggests that she recognises that likening peers to primates is a form of dehumanisation (Haslam, 2006). Black people are often dehumanised by being compared to primates (Owusu-Bempah, 2017). It is noteworthy that Tracie says, ‘but what can I do?’, indicating that she feels powerless.

Students also discussed being likened to primates. For example, Souleymane, a 13-year-old Black African boy with dark skin, said: ‘They call me a Blackie and monkey, most of the people do it here’. Similarly, Sean, a 13-year-old Indian boy with dark brown skin, said: ‘[T]hat happens a lot because people are like, “Come here, you monkey”’.

Several participants said students made slave jokes about Black peers related to skin shade. For example, Mickail, a 17-year-old Black Caribbean young man with dark skin, said that Black peers with light skin call him ‘slave’:

[T]hey were just talking about, ‘Oh yeah, what animal does this person look like?’. Then they got to me and then it wasn’t even an animal, they just said, ‘Slave, slave’. and from there that was just the name they gave me.

Later in the interview he said that they called him ‘slave’ because of his skin shade, suggesting that his peers associated dark skin with transatlantic slaves.

Some participants highlighted jokes about other phenotypical features. For example, a couple said that peers were teased for having Afro hair. When asked whether a peer was teased because of his skin colour, Rochelle said that instead, he was called ‘Nappy Boy’, a pejorative reference to his hair texture. Robinson (2011: 363–364) argues that this texture of hair is ‘the most devalued’, although the term ‘nappy’ has been reclaimed by some Black women (see Rowe, 2019).

Some students said South Asian and other Asian adolescents are teased due to other phenotypical features. For instance, a few described being teased for being hairy. Dilara said:

[A]s a brown girl we do tend to grow a lot of body hair and facial hair quite like naturally and quite fast. [. . .] That was another thing I was bullied for in secondary, because people used to say that I had a moustache and I was a man.

Dilara being called ‘a man’ underlines the fact that hairiness remains taboo for women (Kuipers and van der Ent, 2016). Her narrative speaks to the derogatory popular stereotype about South Asian people being undesirably hairy, something which South Asian comedians have explored in the UK and beyond (e.g. Michael, 2013).

A few Asian participants described how East or Southeast Asian adolescents are mocked for having small eyes. For example, Gabriel, who is Filipino, said: ‘Usually it’s the typical jokes, like for Asians they have small eyes’. Similarly, Giang, a 14-year-old Vietnamese girl with light skin, said: ‘before people were rude to me and they drew like an Asian person with really small eyes’. In research on university students’ perceptions of humour based on racialised and ethnic stereotypes in Hawaii, Caparoso and Collins (2015: 203) found that ‘small eyes’ were in the top 20 ‘frequently recalled stereotypes’ and ‘small eyes’ were perceived to be associated with Asians.

## The politics of colourist jokes

Participants described how colourist jokes were used to establish and test the boundaries of friendships, with rules about who could make colourist jokes to whom. Participants frequently outlined the parameters of permissible colourist joke-making, explaining that the perpetrator’s relationship to the target helped to determine whether jokes would be deemed acceptable. For instance, when asked how he knows when colourist jokes are taken as just jokes and when they have hurt somebody, Gabriel makes a distinction between joking with friends and ‘other people’:

[W]hen I actually tell the joke to my friends, they say, ‘[S]top it now, I think that’s a bit too much’, but we still keep on doing it lightly. If I say that to some other people that it actually offends, you could tell by their reaction [. . .] That’s why I tend not to do it to other people that I’m not very familiar with.

In saying he generally refrains from making colourist jokes about ‘other people’ because they will get offended, but jokes with friends, Gabriel demonstrates that he and his friends have a closer bond. In this way colourist teasing operates like ethnic teasing, which indicates confidence that the relationship between the perpetrator and target can withstand it (cf. Douglass et al., 2016).

Similarly, discussing how it is received when Black people call each other ‘monkey’, Ibraheem said: ‘Well, obviously, because they’re friends, they don’t care, so the person who says it, the other person will probably say it back to them. [. . .] They know that the person doesn’t actually mean what they’re saying.’

This idea that people respond to colourist jokes with a ‘nonserious humour mindset’ (Ford and Ferguson, 2004) when their friends are the perpetrators parallels Benner et al.’s (2024) finding that adolescents are more likely to take racist jokes as a joke when the perpetrator is their friend and more likely to see them as discriminatory when they are perpetrated by someone more distant, particularly if the perpetrator occupies a position of power due to characteristics such as being White and/or popular.

Mickail complicated the idea that colourist jokes are acceptable within friendships:

I feel like they understand the gravity of the situation of what it can cause to somebody, but they feel because they’re your friend that it gives them a kind of pass [. . .] that it [. . .] allows them to say whatever they want to me, because I’m their friend. But, in reality, I’m going to sanction it because I don’t want that type of energy around me or people saying those things. And even, you know, my mum, [. . .] she’ll tell me like [. . .] ‘those aren’t jokes that friends should be making’. So, that kind of just opened my eyes and then I’m now very wary and very observant of who I’m around.

Mickail suggests that his peers know how damaging colourist teasing can be, and yet they persist with it. He suggests that his mother’s explanation of the limits of acceptable joke-making in friendship groups has contributed to his setting boundaries and narrowing his social circle.

Many participants distinguished between minoritised ethnic and White adolescents, arguing that, in most cases, while it might be acceptable for the former to make colourist jokes, it was seldom acceptable for the latter to do so, except in certain friendship groups (cf. Caparoso and Collins, 2015). When Sekani, an 18-year-old Black African young man with dark skin, was asked what if a White person made the same kind of comments that Black people make, such as, ‘You look like a gorilla’?, he responded, ‘Say they’re my friends or something, I wouldn’t really think twice about it. But let’s say if they weren’t my friends, I’d be like that’s a bit dodgy, why are you calling me a gorilla or a monkey?’

Similarly, Alisa, a 14-year-old Mixed (Black-White) girl, described the racialised boundaries around who can make colourist jokes at her school, arguing that it was not permissible for White people to do so:

[N]one of the White people at our school dare say anything because they’ll get jumped or whatever, but still now I’ve noticed that the light people say stuff to the Black people because we’re still Black, so you can say it. [. . .] The Black people say it to each other as well, like dark people.

Her comments fit with Caparoso and Collins’s (2015) argument that humour based on stereotypes is generally seen as offensive when the perpetrator’s focus is on ridiculing a different racialised or ethnic group. While Alisa said that White people in her school would not ‘dare’ to make colourist jokes, she said those with light skin feel entitled to make colourist jokes towards those with dark skin. The fact that colourist humour can be perpetrated by people with light skin who are higher up colourist and racialised hierarchies without being recognised as offensive means that the harm this causes is unacknowledged, and the discrimination is easier to normalise and more difficult to resist.

Several participants suggested that adolescents with dark skin made colourist jokes about peers with dark skin. Rather than this being seen solely as internalised racism, it was described as a pre-emptive strike to give them control of the use of colourist humour and lessen its impact on them by making someone else the initial target. For instance, John, a 14-year-old Mixed (Black-White) boy, said that a person with dark skin will make colourist jokes to someone else with dark skin: ‘[T]hey just say it before the other person can say it back to them’. This suggests that self-deprecating intra-group humour is being used to gain social acceptance by aligning with dominant cultural norms (cf. Guzman, 2020). However, minoritised ethnic people engaging in ‘amused colourist contempt’ in this way also reinforces a ‘white racial frame’ (cf. Pérez, 2022), a term Feagin (2020: 4) coined to describe ‘the dominant racial frame that has long legitimated, rationalized, motivated, and shaped racial oppression and inequality’. While Feagin’s focus was on the USA, this framing also applies to the UK.

Participants also described peers making jokes about their own dark skin. For example, Leon said: ‘[E]veryone was in a room and then he switched off the lights and then he just smiled and then he was like, “Look guys, all you can see is my eyes and teeth”’. The jokes were sometimes characterised as motivated by a desire to protect a person from being the target of colourist jokes. Naomie, a 17-year-old Black African young woman with dark skin, said:

There’s a guy in my form and he’s darker than me, but I can tell – [. . .] maybe if someone was going to say something about him, he would say it before they could say it about him. So, he would be like, ‘Yes, I know I’m dark’. [. . .] Like, he’ll try to get it out before they say it.

The interviewer asked whether Naomie thought that peers would have teased that student if he had not made comments about himself and she said, ‘Yeah, probably’. The interviewer said, ‘So it’s kind of self-protection?’, and Naomie said, ‘Yeah, I think that’s what it is’.

Skye, a 16-year-old Mixed (Asian-Black) young woman with light skin, said that she and her friend liken themselves to primates in a context where there is ‘a lot of teasing going around’ as ‘it’s just our way of, kind of, reclaiming the comments that a lot of people make towards us’. Much has been written about the different ways in which minoritised ethnic people reclaim offensive terms, including the ‘N-word’ (see Popa-Wyatt, 2020). The examples above show that self-deprecating colourist humour can be used as self-defence by striking first and/or attempting to align perpetrators with people higher up the colourist hierarchy (cf. Pérez, 2022). However, when minoritised ethnic people engage in self-deprecating colourist humour it helps to normalise the discrimination, which makes it more insidious.

## The impact of colourist jokes

Participants had contrasting views on the impact of colourist jokes. Some argued that colourist jokes have no impact on those targeted, that they are ‘just jokes’ or ‘banter’ and no one takes offence. However, others suggested that they, or peers, were hurt by colourist jokes, though some said that those targeted sometimes laughed along due to social pressure.

Many participants described colourist jokes as inconsequential. For example, Skye said that Black people made the most colourist jokes and ‘[t]hey were received lightly’ and not taken personally. Similarly, when asked whether he hears students call peers ‘monkey’ at school, Sundeep, a 14-year-old Indian boy with medium skin, said, ‘[I]t’s all jokes’ and ‘No one takes offence to it’.

Some students suggested that the perpetrators of colourist jokes were not seeking to be malicious. For example, Keisha, a 17-year-old Black Caribbean young woman with medium skin, said ‘there is no malice’ behind the jokes:

[I]f you are in a predominantly Black friend group, they will make those kind of inappropriate jokes that I know people like my mum and my dad and everyone else would be like, ‘That is not funny’, [. . .] but [. . .] it is more like friendly fire.

Keisha’s description of colourist jokes as ‘friendly fire’ resonates with Gaut’s (1998) argument that some people view humour as exempt from normal ethical constraints because those employing humour are only joking.

In contrast to those who claimed that colourist jokes had no impact, some students said they were not seen as funny, that they made peers feel bad or hyper aware of their skin shade or other features, which could be isolating. They also said students sometimes felt pressured to laugh along with colourist jokes to downplay the jokes’ effects on them.

When Daashan, a 13-year-old Indian boy with medium brown skin, was asked whether people find it funny when those with dark skin are called ‘monkey’, he suggested that ‘[m]ost of the time, no’, but that the targets can feel too intimidated to challenge the colourist prejudice:

Interviewer: Does anyone intervene and say, ‘Stop, that’s not funny’?

Daashan: No, not really, people don’t really do that as much. They should.

Interviewer: Are people kind of worried to stand up to them?

Daashan: I think so, yeah, because if it’s a lot of people making the jokes and there’s one person, it feels intimidating sometimes.

While the perpetrators of colourist jokes can construct them as ‘harmless’ and just a joke, this can silence or discredit targets when they speak out against colourist humour (cf. Trindade, 2021).

Relatedly, when asked whether he thinks people’s feelings are hurt when colourist jokes are told, Gabriel said: ‘Absolutely’. Similarly, Nimo, a 13-year-old Black African girl with medium skin, suggested that colourist jokes hurt the feelings of the girls subjected to them. This suggests that colourist jokes negatively affect adolescents’ wellbeing in a similar way to racist jokes, the effects of which are often downplayed (Benner et al., 2024).

Asked about the impact of colourist jokes, Ayaan suggested they were alienating and made people with dark skin self-conscious. She said: ‘[I]t makes them feel like more separated because of their skin tone and makes them hyper aware of the fact that they’re darker’. Ayaan explained that her skin shade was ‘pretty similar’ to the targets’, ‘just a tad bit lighter’, which makes her question how she is seen:

[S]ometimes I feel like there’s not that [much] of a difference between me and other darker-skinned students’ skin tones. But then when that kind of stuff happens to them it makes me think [. . .] am I considered that much more light?

Ayaan’s comment that colourist humour is alienating resonates with Peltola and Phoenix’s (2022) findings that humour can other and de-value peers. Ayaan questioning her skin shade because of how those around her are treated suggests that vicarious colourist humour negatively impacts the psychological wellbeing of Black adolescents (cf. English et al., 2020).

For some students, being the target of colourist jokes led to appearance-modifying behaviours in a bid to avoid being stigmatised. For example, Dilara said colourist jokes and bullying about facial hair led her to shave:

I was really young and having to shave and I didn’t really know how to use razors or anything, but I didn’t want people to bully me into doing it. And I know this other guy in my year also had a similar problem, he had like a unibrow and he was also brown, I think he was Pakistani. And people were saying the same thing to him and making fun of it, so he ended up shaving it off. And I think it’s really sad how – what people can say can affect us.

Dilara highlights how colourist teasing led her and her South Asian peer to change their practices. It is poignant that she describes the impact of others as ‘really sad’. Similarly, Ihaan, a 14-year-old Arab boy with medium skin, said: ‘[W]hen I was younger and I used to get mocked about my eyebrows, it still sits with me now. I still think about it. So that’s why I cover my eyebrows.’ Ihaan still seeks to hide part of himself due to colourist teasing as a younger child. Taken together, their narratives suggest that colourist jokes can have long-term effects on girls and boys.

Some participants downplayed the effects of colourist jokes, even as they acknowledged being affected by them. For instance, Imani, a 14-year-old Black African girl with dark skin, said: ‘Obviously, me, I don’t really care. I mean, I care, but I don’t really – it’s not a matter that I would get emotional about something like that. I’ve just taken a joke, as always.’ In this extract, she says she does not ‘really’ care, then says she cares, then denies that she does (‘don’t really’), to downplay the impact of colourist teasing on her and perhaps to avoid constructing herself as a victim.

Similarly, Tiwa, a 13-year-old Black African girl with medium skin, said that the ‘Where did you go?’ colourist joke ‘doesn’t annoy me a lot. It is just kind of a joke. So, I kind of laugh it off because it doesn’t matter that much to me.’ However, when she was asked, ‘Does it annoy you a little bit? And does it matter a bit?’, Tiwa said, ‘Yes, kind of, because if it gets out of hand and it keeps on getting higher and higher, then that’s where it becomes more irritating and annoying’. Like Imani, Tiwa’s initial reluctance to express annoyance at colourist teasing suggests that she may find it more frustrating than she is comfortable admitting.

Others explained that they, or their peers, pretended to find colourist humour funny even though it was hurtful. For example, when asked how being called ‘blackie’ and ‘monkey’ makes him feel, comments that could be read as both colourist and racist, Souleymane said: ‘Not good’. When asked whether people with dark skin ‘play it off as a joke’ when subjected to colourist teasing, he responded: ‘Yeah’. The interviewer then asked, ‘Do you think it has a negative impact on them, or do you think they really do just take it as a joke?’, and he replied: ‘I think they don’t like it’.

Similarly, Tyrone, a 14-year-old Black Caribbean boy with medium skin, responded to a question about how people react to colourist jokes by saying: ‘They always laugh about it, but I know for a fact in some way they feel hurt, so they just try to hide it, I guess’. This suggests that adolescents can feel compelled to adopt a ‘nonserious humour mindset’ (Ford and Ferguson, 2004) when subjected to colourist humour.

Returning to Imani, she suggested that it was easier to laugh along with colourist jokes, than stand up to those making them and risk being subjected to offensive comments:

They were all talking about it, laughing or like, ‘Why is he so dark?’. Giggling. Obviously, I was giggling as well because it’s not – I can’t really say anything, can I? [. . .] If I say, ‘It’s not funny’, the last time that’s what I said. I was like, ‘I can’t lie, it’s not really that funny’. But obviously they’re still going to laugh at that: ‘You’re only saying that because you’re Black’.

Implicit in Imani’s comments is the idea that she laughs at colourist jokes to fit in, aware that speaking out led to her being dismissed and her concerns invalidated: ‘You’re only saying that because you’re Black’. This is consistent with US research which found that the fear of social exclusion can discourage adolescents from speaking out against racialised discrimination or challenging discriminatory behaviour within their peer groups (Mulvey et al., 2016).

For John, the negative impact of colourist jokes was not always instantaneous. When asked how the target of jokes might feel, he said: ‘[S]ometimes they might feel it’s funny but then after it hits them, they feel victimised’. This suggests that some people may laugh along with colourist jokes because they only belatedly appreciate their negative impact.

While many students said those negatively affected by colourist humour did not challenge it, a few said they did speak out and set boundaries to avoid being continually targeted. For example, Mickail, who described being called ‘slave’ by friends (discussed above), said that ‘[a]t first, I thought it was like a joke’. However, he then began to interrogate why he was being called that:

[I]t was because of my skin that they were saying these things and being one of the darkest in the friend group it was like, yeah, no. It’s not going to run. So, I, kind of, just told them how it is, got them all as a collective together. Some took it well, some didn’t, and I was just like, OK, well, if you don’t want to listen then I’ll just move myself away from them.

His comments suggest that his efforts to set boundaries to protect himself from colourist teasing led to his being more discerning about friendships.

For some, participating in the research led to a change in attitude. For example, Danika, a 17-year-old Black Caribbean young woman with dark skin, said that when it came to colourist jokes, she would now:

[N]ot really say those, because obviously they are offensive. Although we’re saying it about ourselves, as well, so it’s kind of like we’re putting ourselves down, even though we’re like having banter with it. But on the grand scale of things it is, kind of, how we perceive ourselves.

As Bemiller and Schneider (2010) argue, jokes communicate both who the perpetrator is and their perceptions of the target(s). If the perpetrator shares disparaged phenotypical features with the target, they are simultaneously mocking themselves when they engage in colourist humour.

## Conclusion: The broader impact of colourist humour

Our article shows that colourist humour reproduces colourist hierarchies in adolescence. We demonstrate that humour is one way in which colourism operates within minoritised ethnic groups, complicating insider/outsider examples of racist joking. We extend theories of ethnic teasing by showing how humour can target subtle differences in appearance (e.g. skin shade) rather than categorical identities (e.g. racialised group), making discrimination easier to normalise and harder to resist. Our intersectional perspective allowed us to attend to how colourist humour operated at school: Black people were the racialised group most often highlighted as making, and being subjected to, colourist jokes, and boys were described as perpetrating, and being subjected to, colourist jokes more than girls. People with dark skin were most often highlighted as the targets of the jokes. These findings reflect the positioning of Black people with dark skin at the bottom of colourist hierarchies (Phoenix and Craddock, 2022), alongside the role of joking as a ‘masculine practice’ that boys have to negotiate (Peltola and Phoenix, 2022: 99).

Our article contributes to the sociology of humour by highlighting the importance of distinguishing between ethnic teasing and racist humour that targets people because of their ethnic/racialised group membership, and colourist humour that focuses on minoritised ethnic people’s skin shade and phenotypical features. Employing the concept of ‘amused colourist contempt’ (cf. Pérez, 2022), we argue that by engaging in colourist humour, young people are reproducing colourist and white supremacist hierarchies, even if they have dark skin or features that do not resemble those associated with whiteness. Many participants described colourist humour as harmful; both for those directly targeted and for onlookers. As Butler (2023: 1) noted, the perception that ‘everything is fair game’ makes humour both alluring and insidious. In our study, while colourist humour was often framed as light-hearted, it simultaneously worked to demean minoritised ethnic adolescents, including those who told jokes themselves.

Given that ‘amused colourist contempt’ reproduces and normalises colourist and white supremacist hierarchies that affect minoritised ethnic adolescents at school and beyond, colourist humour must be treated as a serious issue in schools. Interventions that address colourist humour as a practice that sustains broader systems of inequality are crucial if schools are to become more equitable spaces for young people of all skin shades. Future research should extend this work by examining how adolescents experience colourist teasing online. It would also be valuable to include more South Asian students to deepen understanding of how colourism operates across different groups in the UK. Such research would inform how schools can intervene more effectively. Ultimately, recognising and challenging colourist humour is essential for disrupting the colourist and white supremacist hierarchies it sustains and for creating educational environments where all young people are affirmed and supported.
